# Clinical symptoms and neuroanatomical substrates of daytime sleepiness in Parkinson’s disease

**DOI:** 10.1038/s41531-024-00734-x

**Published:** 2024-08-09

**Authors:** Thaïna Rosinvil, Ronald B. Postuma, Shady Rahayel, Amélie Bellavance, Véronique Daneault, Jacques Montplaisir, Jean-Marc Lina, Julie Carrier, Jean-François Gagnon

**Affiliations:** 1 Center for Advanced Research in Sleep Medicine, CIUSSS-NÎM – Hôpital du Sacré-Coeur de Montréal, Montreal, QC Canada; 2 Department of Psychology, Université de Montréal, Montreal, QC Canada; 3 Research Center, Institut universitaire de gériatrie de Montréal, Montreal, QC Canada; 4 Department of Neurology, Montreal Neurological Institute, McGill University, Montreal, QC Canada; 5 Department of Medicine, Université de Montréal, Montreal, QC Canada; 6 Department of Psychiatry, Université de Montréal, Montreal, QC Canada; 7 Department of Electrical Engineering, École de Technologie Supérieure, Montreal, QC Canada; 8 Centre de Recherches Mathématiques, Université de Montréal, Montreal, QC Canada; 9 Department of Psychology, Université du Québec à Montréal, Montreal, QC Canada

**Keywords:** Parkinson's disease, Human behaviour, Brain

## Abstract

Clinical and neuroanatomical correlates of daytime sleepiness in Parkinson’s disease (PD) remain inconsistent in the literature. Two studies were conducted here. The first evaluated the interrelation between non-motor and motor symptoms, using a principal component analysis, associated with daytime sleepiness in PD. The second identified the neuroanatomical substrates associated with daytime sleepiness in PD using magnetic resonance imaging (MRI). In the first study, 77 participants with PD completed an extensive clinical, cognitive testing and a polysomnographic recording. In the second study, 29 PD participants also underwent MRI acquisition of T1-weighted images. Vertex-based cortical and subcortical surface analysis, deformation-based morphometry, and voxel-based morphometry were performed to assess the association between daytime sleepiness severity and structural brain changes in participants. In both studies, the severity of daytime sleepiness and the presence of excessive daytime sleepiness (EDS; total score >10) were measured using the Epworth Sleepiness Scale. We found that individuals with EDS had a higher score on a component including higher dosage of dopamine receptor agonists, motor symptoms severity, shorter sleep latency, and greater sleep efficiency. Moreover, increased daytime sleepiness severity was associated with a larger surface area in the right insula, contracted surfaces in the right putamen and right lateral amygdala, and a larger surface in the right posterior amygdala. Hence, daytime sleepiness in PD was associated with dopaminergic receptor agonists dosage, motor impairment, and objective sleep measures. Moreover, neuroanatomical changes in cortical and subcortical regions related to vigilance, motor, and emotional states were associated with more severe daytime sleepiness.

## Introduction

Excessive daytime sleepiness (EDS), a marked inability to remain awake and alert during the day^1,2^, is a major non-motor symptom affecting 20–60% of adults with Parkinson’s disease (PD)^3^. While motor and non-motor symptoms in PD tend to coexist and are often related to one another^4^, much less is known about the interdependence between the symptoms associated with EDS in PD. Nonetheless, EDS has been independently but inconsistently linked to various symptoms in PD, such as more severe depressive and anxiety symptoms, worsening motor impairment, and poor quality of life^5–13^. Measures of global cognitive functioning, executive control and processing speed were also reported to be worse in PD adults with EDS than those without^14,15^. As for the interdependence of EDS in PD specifically, two studies have evaluated its interrelation with other motor and non-motor symptoms and provided inconsistent results^16,17^. One study used exploratory factor analysis and found that daytime sleepiness was part of a factor that also included cognitive, autonomic, axial, psychotic and depressive symptoms^17^. In contrast, the other study, using a principal component analysis, found that EDS formed a unique component unrelated to motor and other non-motor symptoms^16^. Further-designed studies are required to understand the interrelations between clinical symptoms in PD. Then, determining whether the clinical manifestation of EDS plays a role in these inter-relational associations between other motor and non-motor symptoms in PD would be easier to establish.

The etiology of EDS in PD is complex and multifactorial^18,19^. EDS in PD is a common adverse effect of dopaminergic therapy (e.g., levodopa or dopaminergic agonists), although some discrepancies in sleep and wakefulness have been shown depending on the dopaminergic agents used^18–21^. Some studies also showed that EDS in PD is associated with complaints of nonrestorative nocturnal sleep^7,8,10,11^. However, most of the studies using nocturnal polysomnography (PSG) showed no group differences nor associations between daytime sleepiness and nighttime sleep efficiency, awakenings, or sleep stages^13,22–25^. Furthermore, the neuroanatomical correlates of EDS in PD are still poorly understood. Only few magnetic resonance imaging (MRI) studies using voxel-based morphometry (VBM) evaluated the gray matter alterations associated with EDS in PD and provided inconsistent results^26–28^. Indeed, some studies have shown a widespread gray matter volume reduction in cortical regions^27,28^, while another have reported increased gray matter volume in the bilateral hippocampal and parahippocampal gyri in PD individuals with EDS compared to those without EDS^26^. A proposed explanation is that VBM relies solely on a volumetric representation of the brain, which can amplify the impact of partial volume and obscure the detection of slight pathological variations in the cerebral cortex and subcortical surface^29^. Other techniques, such as vertex-based cortical and subcortical surface analysis and deformation-based morphometry, may provide a more comprehensive and accurate description of the brain abnormalities associated with EDS in PD.

To further explore the correlates of EDS in PD, we performed two studies. In *Study 1*, we aimed to evaluate the interdependence between clinical symptoms reportedly associated with EDS in PD, and to determine whether the clinical manifestation of EDS could discriminate individuals with PD on the components resulting from this prior assessment. To do this, we evaluated the inter-relations between clinical variables (cognition, neuropsychiatric, motor, dopaminergic medication, sleep-related complaints of insomnia, and PSG sleep measures) using a data-driven approach with a principal component analysis (PCA). Then, we compared PD participants with and without EDS on the resulting components of the PCA. In *Study 2*, we assessed the neuroanatomical substrates underlying daytime sleepiness in PD using surface-, volume- and deformation-based MRI analyses.

## Results

**Fig. 1:**
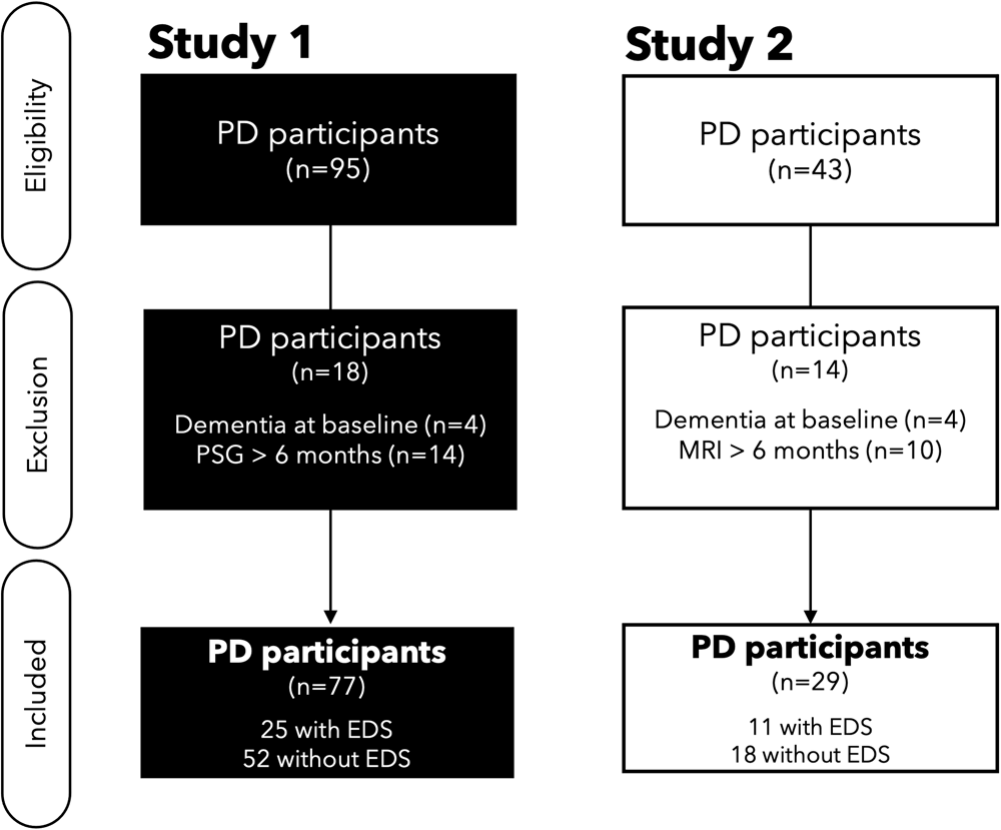
Flowchart of participants included *in Study 1* (black) *and Study 2* (white). For *Study 1*, 95 participants met the inclusion criteria. Of these, 18 were excluded: dementia (*n* = 4) or PSG date > 6 months from the clinical visit (*n* = 14). Then, 77 participants were included in the analyses (25 with and 52 without EDS). For *Study 2*, 43 participants met the inclusion criteria. Of these, 14 were excluded: dementia (*n* = 4) and MRI date > 6 months from the clinical visit (*n* = 10). Then, 29 participants were included (11 with and 18 without EDS). PD Parkinson’s disease, PSG polysomnography, MRI magnetic resonance imaging, EDS excessive daytime sleepiness.

The flowchart for both studies is presented in Fig. 1.

In *Study 1*, individuals with EDS were younger and took a higher dosage of DA receptor agonists (Table 1). No significant between-group differences were found for sex, education, PD duration, disease severity, motor symptom severity, the proportion of individuals with RBD or MCI, MMSE score, LEDD, the proportion of individuals taking DA receptor agonists, antidepressants, other medication, and questionnaires (except ESS). Moreover, the two groups were similar on all PSG variables (Table 2).

**Table 1:** Sociodemographic and clinical characteristics of participants in *Study 1*

Characteristics	PD-nEDS (*n* = 52)	PD-EDS (*n* = 25)	*P*-value
Age, y	66.6 ± 8.3	61.8 ± 8.3	0.02 (0.07)
Sex, male *n* (%)	32 (62)	16 (64)	0.84
Education, y	14.8 ± 4.01	15.0 ± 2.8	0.83
PD duration (symptoms onset), y	5.9 ± 4.5	6.3 ± 4.5	0.74
Hoehn and Yahr stage	2.3 ± .8	2.2 ± 1.0	0.64
UPDRS part III ‘on’ score	21.3 ± 10.7	22.6 ± 10.3	0.62
RBD, n (%)	26 (50)	14 (56)	0.62
MCI, n (%)	25 (48)	14 (56)	0.52
MMSE score	28.5 (1.4)	28.2 (2.2)	0.54
*Medication*			
LEDD, mg	591.4 ± 328.5	629.9 ± 334.5	0.63
DA receptor agonist, *n* (%)^a^	15 (29)	11 (44)	0.21
DA receptor agonist, LEDD mg	55.5 ± 94.6	119.5 ± 170.9	0.04 (0.06)
Antidepressants, *n* (%)	12 (23)	7 (28)	0.27
Others, *n* (%)^b^	13 (25)	6 (24)	0.92
ESS score	6.1 ± 2.8	14.20 ± 3.06	<0.0001 (0.64)
ISI score	10.1 ± 7.0	12.6 ± 6.5	0.14
BDI-II score	10.4 ± 7.8	11.8 ± 5.3	0.42
BAI score	11.1 ± 8.3	9.9 ± 7.0	0.53
UPDRS part I apathy, n (%)	17/47 (36)	12/24 (50)	0.26

Results are expressed as mean ± standard deviation (Effect size when *p* < 0.05).

*PD* Parkinson’s disease, *PD-EDS* PD with excessive daytime sleepiness, *PD-nEDS* PD without EDS, *DA* dopamine, *UPDRS* Unified Parkinson’s Disease Rating Scale, *LEDD* levodopa equivalent daily dosage, *MCI* mild cognitive impairment, *MMSE* mini-mental state examination, *RBD* rapid eye movement sleep behavior disorder, *ESS* Epworth Sleepiness Scale, *ISI* Insomnia Severity Index, *BDI-II* Beck Depression Inventory second edition, *BAI* Beck Anxiety Inventory.

^a^All on pramipexole.

^b^Clonazepam = 8, zopiclone = 5, lorazepam = 2, quetiapine = 1, tranxene = 1, oxazepam 1, and nitrazepam 1.

**Table 2:** PSG variables in PD participants with and without EDS in *Study 1*

PSG variables	PD-nEDS (*n* = 52)	PD-EDS (*n* = 25)	*P*-value
Total sleep time, min	333.8 ± 111.3	381.8 ± 71.4	0.32
Sleep latency, min^a^	21.8 ± 25.9	11.7 ± 14.0	0.33
WASO, min	138.6 ± 85.0	98.0 ± 63.3	0.29
Sleep efficiency, %	72.4 ± 18.7	81.5 ± 11.7	0.22
Wake, %	27.6 ± 18.7	18.5 ± 11.7	0.22
N1, %	18.0 ± 11.0	17.4 ± 8.7	0.81
N2, %	58.8 ± 11.2	59.5 ± 11.3	0.60
N3, %	6.8 ± 7.9	5.3 ± 7.2	0.44
REM sleep, %	17.0 ± 9.3	19.3 ± 7.3	0.30
REM sleep latency, min	166.2 ± 97.9	153.7 ± 81.8	0.60
AHI^a^	7.0 ± 8.7	6.8 ± 6.2	0.62
Mean O_2_ saturation	94.9 ± 1.7	95.7 ± 1.4	0.19
Minimum O_2_ saturation	89.1 ± 4.1	90.6 ± 3.4	0.11
Microarousal Index	14.8 ± 7.6	17.3 ± 6.0	0.15

Results are expressed as mean ± standard deviation (Effect size when *p* < 0.05).

Since age and DA agonist (LEDD, mg) differed significantly between the two groups (*p* < 0.05), an analysis of covariance (ANCOVAs) was performed on each PSG variable using age and DA agonist (LEDD, mg) as covariables.

*PD* Parkinson’s disease, *PD-EDS* PD with excessive daytime sleepiness, *PD-nEDS* PD without EDS, *PSG* polysomnographic, *DA* dopamine, *LEDD* levodopa equivalent daily dosage, *WASO* wake after sleep onset, *N1* non-rapid eye movement Sleep Stage 1, *N2* non-rapid eye movement Sleep Stage 2, *N3* non-rapid eye movement Sleep Stage 3. *REM* rapid eye movement.

^a^Log transformed.

In *Study 2*, individuals with EDS took a higher dosage of DA receptor agonists (Table 3). Age, sex, education, PD duration and disease severity, motor symptoms severity, proportion of individuals with RBD or MCI, MMSE score, LEDD, proportion of individuals taking DA receptor agonists, antidepressant, others medication and questionnaires (except ESS) were similar between groups.

**Table 3:** Sociodemographic and clinical characteristics of participants in *Study 2*

Characteristics	PD-nEDS (*n* = 18)	PD-EDS (*n* = 11)	*P*-value
Age, y	65.7 ± 9.5	60.8 ± 9.6	0.19
Sex, male, n (%)	11 (61.1)	6 (54.5)	0.73
Education, y	14.3 ± 3.9	15.1 ± 3.3	0.60
PD duration (symptoms onset), y	7.1 ± 5.9	6.9 ± 5.7	0.93
Hoehn and Yahr stage	2.3 ± 0.8	2.3 ± 1.0	0.99
UPDRS part III ‘on’ score	23.7 ± 9.3	20.8 ± 9.7	0.43
RBD, n (%)	11 (61.1)	6 (54.5)	0.59
MCI, n (%)	9 (50)	6 (54.5)	0.81
MMSE score	28.5 (1.4)	27.9 (3.0)	0.48
Medication			
LEDD, mg	562.5 ± 390.3	640.9 ± 328.0	0.58
DA receptor agonist, n (%)^a^	6 (33)	6 (55)	0.26
DA receptor agonist, LEDD mg	65.8 ± 115.1	190.9 ± 202.3	0.04 (0.14)
Antidepressants, n (%)	6 (33)	3 (27)	0.66
Others, n (%)^b^	4 (22)	1 (9)	0.36
ESS score	6.6 ± 2.5	12.5 ± 1.6	<0.0001 (0.66)
ISS score	8.9 ± 5.7	12.3 ± 7.2	0.20
BDI-II score	9.8 ± 6.0	11.9 ± 5.9	0.38
BAI score	10.6 ± 9.4	7.5 ± 8.3	0.38
UPDRS part I apathy, n (%)	5/17 (29)	5/11 (45)	0.39

Results are expressed as mean ± standard deviation (Effect size when *p* < 0.05).

*PD* Parkinson’s disease, *PD-EDS* PD with excessive daytime sleepiness, *PD-nEDS* PD without EDS, *DA* dopamine, *UPDRS* Unified Parkinson’s Disease Rating Scale, *LEDD* levodopa equivalent daily dosage, *MCI* mild cognitive impairment, *MMSE* mini-mental state examination, *RBD* rapid eye movement sleep behavior disorder, *ESS* Epworth Sleepiness Scale, *ISI* Insomnia Severity Index, *BDI-II* Beck Depression Inventory second edition, *BAI* Beck Anxiety Inventory.

^a^Pramipexole = 11 and ropinirole = 1.

^b^Clonazepam = 4 and quetiapine = 1.

For *Study 1*, PCA analyses revealed six components from the fourteen variables, and the resulting factor structure explained 68.3% of the variables’ variance. The six components and their loadings are listed in Table 4.

**Table 4:** Summary of the principal component analysis

Components items/variables	1	2	3	4	5	6
PD duration (symptoms onset), y				0.53		
DA receptor agonist, LEDD mg	0.60					0.42
UPDRS part III “on” score	0.42	0.46				−0.43
BDI-II score			0.88			
BAI score			0.72			
ISI score					0.59	
Sleep latency (min)^a^	−0.75					
Sleep efficiency (%)	0.71					
N2-N3 sleep (%)				0.80		
AHI					−0.85	
Mean O_2_ saturation (%)						0.80
MMSE, score		−0.79				
TMT B, time		0.75				
Bells Test, number of omissions		0.46		−0.52		
% variance	20.3	13.0	11.5	8.0	7.8	7.6
Eigenvalues	2.85	1.82	1.62	1.12	1.10	1.07

*PD* Parkinson’s disease, *DA* dopamine, *LEDD* levodopa equivalent daily dosage, *UPDRS* Unified Parkinson’s Disease Rating Scale, *BDI-II* Beck Depression Inventory second edition, *BAI* Beck Anxiety Inventory, *ISI* Insomnia Severity Index, *N2* non-rapid eye movement Sleep Stage 2, *N3* non-rapid eye movement Sleep Stage 3, *AHI* Apnea-Hypopnea Index, *MMSE* mini-mental state examination, *TMT B* Trail Making Test part B.

^a^Log transformed.

Component 1 accounted for 20.3% of the total variance and included four variables: *DA receptor agonists dosage*, *UPDRS-III, sleep latency*, and *sleep efficiency*. Component 2 accounted for 13.0% of the total variance and was composed of four variables: *UPDRS-III, MMSE*, *Trail Making Test B*, and *Bells Test*. Component 3 accounted for 11.5% of the total variance and comprised two variables: *BDI-II* and *BAI*. Component 4 accounted for 8.0% of the total variance and included three variables: *PD duration*, *N2-N3 sleep*, and *Bells Test*. Component 5 accounted for 7.8% of the total variance and included two variables: *ISI* and *AHI*. Component 6 accounted for 7.6% of the total variance and comprised three variables: *DA receptor agonists dosage*, *UPDRS-III*, and *mean O*_*2*_ *saturation*.

**Fig. 2:**
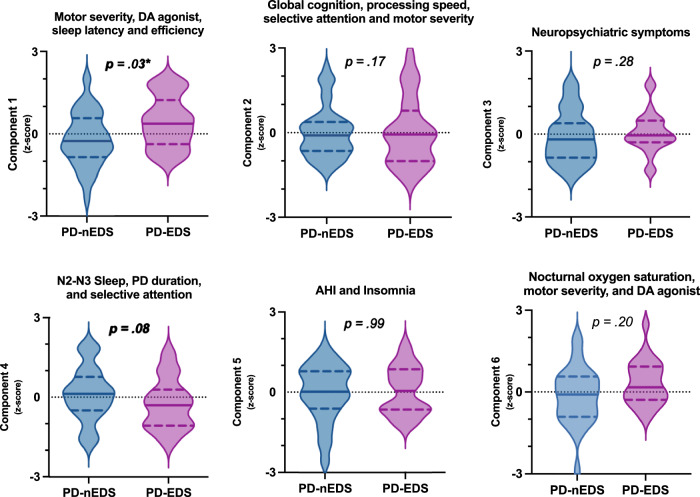
Principal component analysis expressed in z-scores are depicted on *Y*-axis. The full line in each violin plot represents the median and the dotted line represents the first and the third quartile. *Individuals with EDS had a higher score on component 1 as compared to those without EDS (*F* = 4.66, *p* = 0.03, *ES* = 0.06; PD-nEDS: *M* = −0.21, *SD* = 0.97; PD-EDS: *M* = 0.43, *SD* = 0.94). A statistical trend was found for component 4 (*F* = 3.11, *p* = 0.08, *ES* = 0.04; PD-nEDS: *M* = 0.12, *SD* = 1.01; PD-EDS: *M* = −0.26, *SD* = 0.95), whereas no difference was found for components 2 (*F* = 1.92, *p* = 0.17, *ES* = 0.03; PD-nEDS: *M* = 0.00, *SD* = 0.91; PD-EDS: *M* = −0.00, *SD* = 1.19), 3 (*F* = 1.17, *p* = 0.28, *ES* = 0.02; PD-nEDS: *M* = −0.12, *SD* = 0.92; PD-EDS: *M* = 0.06, *SD* = 0.74), 5 (*F* = 0.00, *p* = 0.99, *ES* = 0.00; PD-nEDS: *M* = −0.02, *SD* = 1.05; PD-EDS: *M* = 0.05, *SD* = 0.90), and 6 (*F* = 1.65, *p* = 0.20, *ES* = 0.02; PD-nEDS: *M* = −0.15, *SD* = 1.05; PD-EDS: *M* = 0.32, *SD* = 0.81). PD Parkinson’s disease, PD-EDS PD with excessive daytime sleepiness, PD-nEDS PD without EDS, AHI apnea-hypopnea index, DA dopaminergic agonist, M mean, SD standard deviation, ES effect sizes.

A group difference was found for component 1 (Fig. 2). Compared to individuals without EDS (*M* = −0.21, *SD* = 0.97), those with EDS (*M* = 0.43, *SD* = 0.94) had a higher component 1 score that included higher dosage of *DA receptor agonists (LEDD in mg)*, higher *UPDRS-III* scores, shorter *sleep latency*, and greater *sleep efficiency*. A statistical trend was also observed for component 4. No significant between-group difference was found for components 2, 3, 5, and 6.

**Fig. 3:**
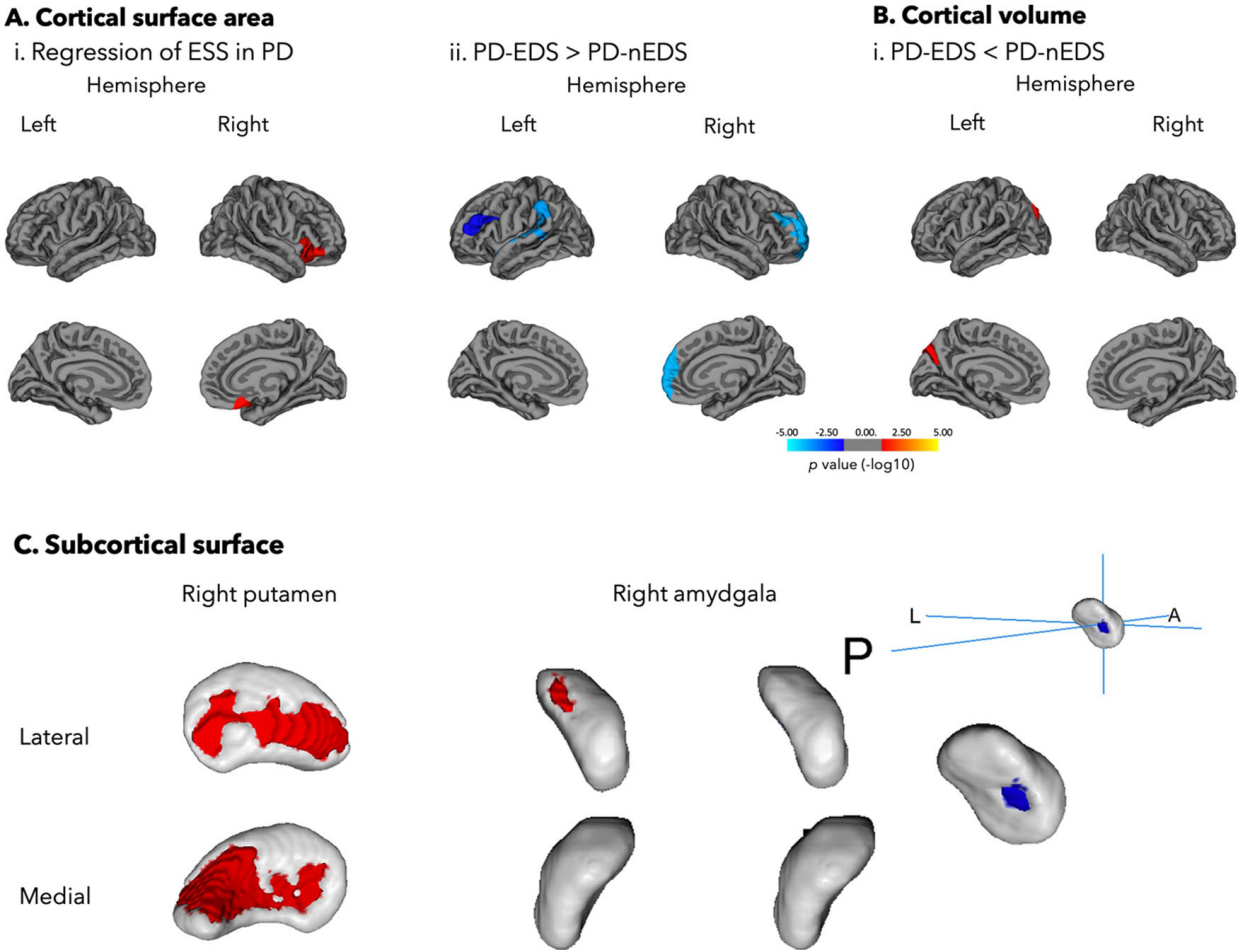
Cortical and subcortical changes associated with daytime sleepiness and EDS in PD individuals. **A** (i). A higher ESS score was associated with larger surface area in the right anterior insula extending to the medial orbitofrontal cortex. (ii). Individuals with EDS had larger surface area in the right rostral middle frontal cortex, left supramarginal cortex extending to the posterior insula and superior temporal gyrus and left caudal middle frontal area. **B** Individuals with EDS had decreased cortical volume in the left parieto-occipital sulcus including the fundus. The color bar indicates the logarithmic scale of *p* values (−log10) for between-group differences, with red-yellow areas representing reductions in the first compared to the last group in the contrast (corrected with Monte Carlo simulation at *p* < 0.05 with age, sex, and disease duration as covariates as well as total intracranial volume for cortical volume analysis). **C** A higher ESS score was associated with contraction (red) in the lateral and medial surface of the right putamen, contraction in the posterior surface of the right amygdala, and expansion (blue) in the posterior surface of the right amygdala. ESS Epworth Sleepiness Scale, PD-EDS Parkinson’s disease with excessive daytime sleepiness, PD-nEDS PD without EDS.

In *Study 2*, for vertex-based cortical surface in PD, higher ESS scores were associated with larger surface area in the right anterior insula extending to the medial orbitofrontal cortex (Fig. 3a and Table 5). Moreover, between-group differences revealed that individuals with EDS had larger surface area in the right rostral middle frontal cortex, left supramarginal cortex extending to the posterior insula and superior temporal gyrus and left caudal middle frontal area. Individuals with EDS also had decreased cortical volume in the left parieto-occipital sulcus including the fundus (Fig. 3b and Table 5).

**Table 5:** Cortical and subcortical abnormalities in PD participants with EDS and without EDS

Cortical metric	Region	Hemisphere	Cluster size, mm^2^	No. of vertices	Talairach coordinates			−log10 *p* value
					*x*	*y*	*z*	
A. *Regression of ESS*								
Cortical surface area	Insula	R	2058.91	4354	29.9	20.1	4.6	2.829
B. *PD-EDS* *>* *PD-nEDS*								
Cortical surface area	Rostral middle frontal	R	4069.27	5949	25.2	41.8	15.1	−2.909
	Supramarginal	L	3525.14	8516	−57	−41.1	35.1	−2.927
	Caudal middle frontal	L	2076.84	3515	−34.2	17.3	22.4	−3.687
C. *PD-EDS* *<* *PD-nEDS*								
Cortical volume	Precuneus	L	1523.91	2538	−15.5	−66.7	36	3.441

Subcortical metric	Region	Hemisphere	No. of vertices	MNI152 coordinates		
				*x*	*y*	*z*
D. *Regression of ESS*						
Contraction	Putamen	R	1239	19	11	0
	Amygdala	R	40	30	−9	−16
Expansion	Amygdala	R	44	28	−7	−23
E. *PD-EDS* *<* *PDnEDS*						
	Putamen	R	455	19	6	−7
F. *PD-EDS* *>* *PD-nEDS*						
	Amygdala	R	19	28	−8	−22

*ESS* Epworth Sleepiness Scale, *PD-EDS* Parkinson’s disease with excessive daytime sleepiness, *PD-nEDS* PD without EDS, *R* right, *L* left.

As for the vertex-based subcortical shape in PD, higher ESS scores were associated with contraction in the lateral and medial surface of the right putamen (*r* = −0.41, *p* = 0.029) and the lateral surface of the right amygdala (*r* = −0.61, *p* = 0.001), and expansion in the posterior surface of the right amygdala (*r* = 0.57, *p* = 0.001) (Fig. 3c and Table 5). Moreover, individuals with EDS show surface contraction of the right putamen and surface expansion on the right amygdala compared to those without EDS.

No significant association with ESS scores or group differences were found for whole-brain analyses with cortical thickness, VBM or DBM techniques.

## Discussion

This article presents two studies conducted to (1) clarify the association between daytime sleepiness and PD after computing a PCA to create components of other clinical motor and non-motor symptoms, and (2) determine the neuroanatomical changes associated with daytime sleepiness in PD. In *Study 1*, each component obtained from the PCA defined a putative discriminant combination of variables to contrast PD individuals based on EDS (defined as a score >10 on the ESS). The results highlight that the combination of a higher dosage of DA receptor agonists, more severe motor symptoms, shorter sleep latency, and greater sleep efficiency can distinguish PD individuals with EDS from those without. In *Study 2*, the results show that more severe daytime sleepiness in PD is associated with increased cortical surface area (right anterior insula) and subcortical surface contraction (right putamen and right amygdala). These regions are associated to vigilance, motor, and emotional states. Together, these two studies provide new insight to better understand the complex relationships between daytime sleepiness and the other clinical symptoms, as well as structural brain changes reported in PD.

With the magnitude of symptoms that may or may not be independently involved in daytime sleepiness in PD^5–13^, it becomes necessary to provide an approach allowing both data reduction while also evaluating the interrelations between these symptoms. Here, *Study 1* offers a sensitive picture of the relationship between clinical symptoms related to daytime sleepiness in PD. Using a PCA, we propose an alternative strategy to establish the interdependence of clinical factors associated with daytime sleepiness in PD first. Our study highlights the importance of including objective sleep variables to assess the interdependence of clinical symptoms in PD, contrary to similar studies that have solely used questionnaire-based sleep measures^16,17^. Nocturnal sleep metrics (sleep latency, sleep efficiency, N2-N3 sleep, AHI) and variables associated with parkinsonism (dosage of *DA receptor agonists*, PD duration, and severity of motor symptoms) were found within most components of the PCA, making them non-negligible features in PD.

The results highlight that PD individuals with EDS differ from those without EDS on a component including higher DA receptor agonists dosage, more severe motor symptoms, shorter sleep latency, and greater sleep efficiency. Of note, *Study 1* illustrates that the presence of EDS is significant not only for the component with the greatest variance but also for objective sleep variables (sleep latency and efficiency) that were not previously described by other studies^16,17^. In *Study 1*, participants taking a DA receptor agonist were exclusively on pramipexole, which has a high affinity with D2/D3 receptors. This medication has been shown to induce somnolence and daytime sleep episodes in PD^19,30^. It suggests that a higher dose of pramipexole to treat the worst motor symptoms can also concomitantly shorten sleep latency and increase sleep efficiency due to its sedative nature. A double-blind placebo-controlled study performed in healthy adults also found that pramipexole administration significantly reduced mean sleep latency and increased total sleep duration on the Multiple Sleep Latency Test^31^. On the other hand, most studies using nocturnal PSG reported no associations between daytime sleepiness and most PSG variables in PD^13,22–25^. One of them, however, reported that PD individuals with EDS had shorter sleep latency compared to those without EDS^13^. While shorter sleep latency and greater sleep efficiency are included in the most discriminative component in *Study 1*, one must remember that it is the component considering the interrelation between all four variables that differ between PD individuals with and without EDS.

The two PD groups did not differ on neuropsychiatric symptoms including anxiety and depression (component 3), and on motor severity, global cognition, executive control and processing speed (component 2). This result is in line with a previous study in PD that also used a PCA^16^; they showed that EDS was not interrelated to anxiety, depression, or global cognition^16^. Moreover, PD individuals with and without EDS did not differ on insomnia severity and AHI (component 5), and on nocturnal oxygen saturation, motor severity and DA receptor agonists dosage (component 6). These results corroborate previous studies, which found no significant association between daytime sleepiness and objective measures related to sleep apnea in PD^13,32–34^.

As for *Study* 2, we applied surface-based analyses of cortical and subcortical structures to allow a more exhaustive picture of daytime sleepiness’s structural brain correlates in PD. Increased daytime sleepiness was associated with a larger surface area in the right anterior insula extending to the medial orbitofrontal cortex. Between-group comparisons also revealed a larger surface area in PD individuals with EDS in the right rostral middle frontal cortex, left supramarginal cortex extending to the posterior insula and superior temporal gyrus and left caudal middle frontal area, and reduced cortical volume in the left parieto-occipital sulcus. The insula is a cortical region highly interconnected with several cortical (frontal, temporal, parietal, occipital, limbic areas) and subcortical (putamen, thalamus, amygdala, and hippocampus) regions^35,36^. It plays an important part in multiple brain networks involved in a wide variety of functions including sensorimotor, olfacto-gustatory, socio-emotional, and cognition^37^. In particular, it is suggested that the insular cortex plays a role in the salience network and is associated with subjective sleepiness^38–41^. Thus, the insula is considered as a central hub processing relevant information related to vigilance, motor responses, and emotional states^37,40–43^. In PD, neuroimaging studies have revealed functional and structural alterations of the insula which would play a potential role in non-motor symptoms reported in this population^40^. Interestingly, increased surface area has been reported in isolated REM sleep behavior disorder^44^, a strong prodromal phenotype associated with the development of PD^42^, and shown to occur preferentially within regions with a higher expression of genes involved in the inflammatory response^43^. Increased area may therefore be consequential to ongoing inflammatory response in the brain. Otherwise, local surface area has been hypothesized to reflect underlying white matter fibers^45^. Increased surface area could result from underlying white matter degeneration since the tension or shrinkage of white matter fibers could lead to deeper sulci and extended cortical surface area^45,46^. Importantly, previous research reported white matter alterations in PD individuals with EDS, which may underlie anterior insula extending to the medial orbitofrontal cortex. Hence, a previous study in PD individuals with EDS reported bilateral white matter damage (illustrated by increased axial diffusivity) notably in the superior corona radiata and the temporal part of superior longitudinal fascicles^26^, as these pass underneath the structures shown in *Study 2*. Microstructural changes of white matter in sleep-related circuits (particularly bilateral fornix and bilateral inferior longitudinal fasciculus) have also been observed in drug-naïve PD individuals with EDS compared to those without EDS^47^. Future multimodal neuroimaging studies using both vertex based, white-matter integrity metrics, and should validate this result interpretation.

Increased daytime sleepiness were also associated with surface contraction of the right putamen and right lateral amygdala, as well as a surface expansion of the posterior surface of the right amygdala. These alterations were also found in PD individuals with EDS (contraction of right putamen, and expansion of right amygdala) compared to those without EDS. Since the right putamen and amygdala are highly inter-interconnected with the right insula^40^, these results could reflect alterations in the mesocorticolimbic circuitry in PD individuals with more severe daytime sleepiness. Putamen atrophy is a frequent feature reported in PD^48,49^. One study, however, reported a localized pallidum and putamen volume hypertrophy, in the left dorsolateral and the right dorsal, respectively, for drug-naïve PD individuals with EDS, and no significant group differences were found in the shape analysis of the other subcortical nuclei^50^. While the latter is contrary to our results, their participants were drug-naïve and in an early stage of PD. Here, participants in *Study 2* were more advanced in their disease progression. Our results imply that putamen and amygdala atrophy could occur as the disease progresses. Accordingly, association of daytime sleepiness with nigrostriatal dopaminergic degeneration, namely for the putamen, was reported in PD individuals at stage 2 of the Hoehn and Yahr scale, but not in PD individuals in stage 1 using SPECT^51^.

No significant association with daytime sleepiness was found using VBM and DBM techniques. To date, the studies that used VBM to evaluate the structural correlates of sleepiness in PD provided inconsistent results^26–28^. One found a widespread gray matter volume reduction in cortical regions (frontal, temporal, occipital, and limbic), in addition to atrophy of the basal forebrain in PD individuals with EDS as compared to those without EDS and healthy controls^27^. Another study, with a larger sample size, had similar results^28^. They identified in PD a loss of integrity and atrophy in the anterior cingulate network that were associated with EDS^28^. The other study reported increased gray matter volume in the bilateral hippocampal and parahippocampal gyri in PD individuals with EDS compared to those without EDS^26^. However, most studies did not control for multiple comparisons (only one did^28^). There has been no study to date evaluating changes in brain morphology related to EDS using DBM in PD.

These two studies have some limitations. Conventional approaches in research usually assess symptoms independently, which provides key information on the bidirectional link between the two variables studied. However, the clinical perspective and reality of PD individuals are rather complex phenomena, and they imply an interplay between clinical symptoms^4^. A conventional approach infers that symptoms in PD are mutually exclusive and rules out the assumption that many symptoms in PD are interrelated to each other. While the approach with a PCA considers this interrelation between clinical symptoms linked to daytime sleepiness in PD and provides additional clinical information, challenges in interpretation and limitations can arise. Of note, it is essential to mention that the PCA method used in *Study 1* is a data-driven approach, and to maintain enough statistical power, we were limited to 14 variables. Furthermore, given the complexity of the protocol (nighttime PSG, neuropsychology, MRI), we cannot exclude bias in selecting our sample (e.g., interest to partake in sleep study due to personal sleep complaints, or participants with advanced PD would be less inclined to embark on our research protocol). Future studies should evaluate whether our conclusions apply to a larger and more representative sample with a wider range of variables. It should also be noted that daytime sleepiness is a subjectively-rated symptom that individuals often underrecognize^23^; caregiver input would have been valuable but was not always available. Another alternative for future studies would be the inclusion of objective measures that are sensitive to the measurement of EDS in PD. Given the strong association between the use of pramipexole and daytime sleepiness in PD, it could be of interest to evaluate whether the timing of pramipexole intake (e.g. evening administration) is associated with shorter sleep latency/greater sleep efficiency and with daytime sleepiness complaints. Moreover, as this study excluded participants with dementia, the assessment of correlations between cognition and daytime sleepiness is limited to mild cognitive changes. As for *Study 2*, group analyses were considered complimentary due to the limited sample size. Although participants in *Study 2* were carefully selected to ensure minimizing selection biases and controlling for several confounding variables, and being stricter in terms of multiple comparisons, a larger sample could increase the statistical power and allow the identification of other brain region changes related to the severity of daytime sleepiness in PD. Nevertheless, future studies could use the data provided here for a priori power analysis and have an accurate required sample size.

## Methods

### Participants

PD participants were recruited from the movement disorders clinics of the *Centre Hospitalier de l’Université de Montréal* and the McGill University Health Centre (Montreal, Canada) as part of a longitudinal study on sleep and cognition in PD. They were consecutive individuals seen at their annual assessment and were referred by a neurologist for this study regardless of sleep complaints including daytime sleepiness. All participants underwent one night in the sleep laboratory at the Centre for Advanced Research in Sleep Medicine of the *Centre Intégré universitaire de santé et de services sociaux du Nord-de-l’Île-de-Montréal* (CIUSSS-NÎM)—*Hôpital du Sacré-Cœur de Montréal*. Research protocols were approved by the ethics committee of the CIUSSS-NÎM *– Hôpital du Sacré-Cœur de Montréal* and by the ethics committee CIUSSS *du Centre-Sud-de-l’Île-de-Montréal*—*Comité d’éthique de la recherche vieillissement-neuroimagerie* (Montreal, Quebec). All participants gave their informed written consent to participate.

For *Study 1*, PD participants underwent multiple clinical visits including a nocturnal PSG, neurological exam, a complete neuropsychological assessment, as well as sleep and mood questionnaires. Participants between 45 and 85 years old with a diagnosis of idiopathic PD confirmed by a movement disorder specialist were included^52^. Exclusion criteria were: (i) dementia according to the neuropsychological assessment^53^, or the recommendations of the Movement Disorder Society Task Force for PD^54^; (ii) a major psychiatric disorder (including bipolar disorder, untreated major depression, schizophrenia) according to the criteria of the Diagnostic and Statistical Manual of Mental Disorders, Fourth Edition, Text Revision^53^; (iii) history of stroke, or cerebrovascular disease; and (iv) PSG date > 6 months from the clinical visit. They were taking their usual medication during the study. Antiparkinsonian medication, including dopamine (DA) receptor agonists, was converted into levodopa equivalent daily dose (LEDD mg) according to Tomlinson et al. ^55^. Motor symptom severity was evaluated by a neurologist in the “on” state using the Unified Parkinson’s Disease Rating Scale, Part III (UPDRS-III)^56^. The Hoehn and Yahr scale was also used to assess disease severity^57^.

For *Study 2*, PD participants underwent the same protocol as *Study 1* in addition to an MRI. Similar inclusion and exclusion criteria were retained from *Study 1* apart from the PSG date criterion since it was not included in the MRI analyses. All participants of *Study 2* also had to have an MRI scan < 6 months from the clinical visit.

### Procedure

Polysomnography (PSG)recordings included EEG (10-20 system, referential montage with linked ears), chin electromyogram and left and right electrooculography. Signals were digitalized at a sampling rate of 256 Hz using commercial software (Harmonie, Stellate System). Sleep stages were identified according to the American Academy of Sleep Medicine criteria^58^. Oral and nasal airflow plus thoracic and abdominal movements were recorded in concomitance with pulse oximetry was performed to measure the respiratory event index. Apneas were defined as an airflow reduction of ≥90% lasting ≥10 s. Hypopneas were defined as an airflow reduction of ≥30% lasting ≥10 s accompanied by either an oxygen desaturation of ≥ 3% or arousal, as recommended^58^. The diagnostic criteria for REM sleep behavior disorder (RBD) were: REM sleep without atonia defined as tonic EMG activity >30% and/or phasic EMG activity >15% of the total REM sleep time (on 3-s mini-epochs within 30-s REM sleep epochs) and at least one of the following two criteria: 1) a history of undesirable and potentially harmful behaviors during sleep or 2) complex behaviors during REM sleep recorded during the night spent in the laboratory^59–61^.

Both studies offered PD participants a comprehensive neuropsychological assessment measuring five cognitive domains: attention, executive functions, verbal episodic memory and learning, visuospatial, and language abilities (see Table S1). Cognitive status was determined by consensus between the neurologist (R.B.P.) and neuropsychologist (J.F.G.). Mild cognitive impairment (MCI) diagnosis was based on specific criteria detailed elsewhere^62,63^. The Mini-Mental State Examination (MMSE) was also completed as a measure of global cognitive functioning^64^. Self-reported questionnaires were used to assess the severity of depressive (Beck Depression Inventory second edition (BDI-II)^65^), anxiety (Beck Anxiety Inventory (BAI)^66^), insomnia (Insomnia Severity Index (ISI)^67^), and apathy (proportion of individuals with a score ≥1 on the UPDRS Part I item 4^56^) symptoms. The Epworth Sleepiness Scale (ESS) was used to evaluate the severity of daytime sleepiness symptoms^68^.

In *Study 2*, PD participants were imaged on a Siemens 3 T TrioTIM scanner with a 12-channel head matrix coil at the *Institut universitaire de gériatrie de Montréal*. T1-weighted images were acquired using an MPRAGE sequence with the following parameters: repetition time 2.3 seconds, echo time 2.91 ms, inversion time 900 milliseconds, 9-degree flip angle, 256 × 240 mm field of view, 256 × 240 matrix resolution (voxel size: 1 × 1 × 1 mm), and 240 Hz/Px bandwidth.

Vertex-based surface analyses were performed to investigate the local changes occurring in the cortical and subcortical surfaces. Cortical surface processing was conducted with FreeSurfer (version 6.0.0) using default parameters, as described previously^44,69^, which generated cortical surface maps that quantified thickness, surface area, and volume at each vertex. Thickness and surface area maps were smoothed with a filter of full width at half maximum of 20 mm and volume maps of 15 mm. Subcortical surface processing using FSL-FIRST was then performed to study the surface of the left and right putamen, caudate, pallidum, thalamus, hippocampus, amygdala, and nucleus accumbens^70^. Briefly, the structures were segmented and inflated based on shape and intensity information from 336 manually delineated T1-weighted images^70^. Surfaces were registered to the MNI152 space and then used to detect between-group differences in surface displacement^71^.

We additionally performed VBM and DBM in CAT12 version 12.7 to describe the volume- and deformation-based changes found in the gray matter tissue of participants. Normalized modulated images were smoothed using a filter of 8 mm and analyzed using default parameters, as described previously^72^.

### Statistical analyses

All variables were examined for their mean, standard deviation, skewness, and kurtosis. Logarithmic transformations (PSG values when applicable) were applied for variables that were substantially out of a normal distribution (i.e., absolute skew value larger than 2 or an absolute kurtosis larger than 7^73^). Non-parametric equivalent tests (demographic and clinical data) were performed when variables were not normally distributed. Statistical analyses were performed using SPSS (version 29) and Matlab (9.6.0) for multiple imputations processing.

For *Study 1*, we performed a PCA using varimax rotation. Due to the sample size of *Study 1*, we limited the number of variables included in the PCA. We thus selected 14 a priori variables based on the literature that were shown to be linked to the presence of EDS in PD and that were available in our study^14,15,18,19^: (1) *PD duration* starting from symptoms onset (years); (2) *DA receptor agonists* dosage converted in LEDD (mg)^55^; (3) motor symptoms severity: *UPDRS-III* total score « on »; (4) depressive symptom severity: *BDI-II* total score; (5) anxiety symptoms severity: *BAI* total score; (6) insomnia symptom severity: *ISI* total score; (7) *sleep latency* (min; log-transformed); (8) *sleep efficiency* (%); (9) *N2 and N3 sleep* (%); (10) *AHI* (log-transformed); (11) *mean O*_*2*_ *saturation* (%); (12) a global cognitive measure: *MMSE* raw score; (13) executive control and processing speed measure: *Trail Making Test, part B* (time); and (14) visuospatial selective attention measure: *Bells Test* (number of omissions).

We selected the 14 a priori variables to be included in the PCA model. Since this selection showed missing data (e.g., incomplete questionnaires), we proceeded to multiple imputation (MI) targeted for exploratory factor analyses as described in Rubin et al. to optimize the sample in the context of limited statistical power^74^. Further details are described in Supplementary Material (Table S2). Once the MI procedure had been completed, we repeated our statistical analyzes with imputed data, and the effects remained similar.

For the Principal Component Analysis (PCA), this factor analysis created components of variables that were highly intra-correlated but not inter-correlated from one another. Each variable included in a component has a loading which characterizes the magnitude of covariance observed between the variables within the same component. The number of factors was determined using a minimal eigenvalue of 1. Eigenvalues, used as references, were derived from random data sets resulting from running 1000 iterations.

PD participants were divided into two groups based on the presence or absence of EDS via a score higher than 10 on the ESS (PD with EDS > 10, PD without EDS ≤ 10). To evaluate potential confounding variables such as age, sex, educational level and RBD status, one-way analysis of variance (ANOVA) comparing PD participants with and without EDS on these variables were first computed. Only age differed significantly between the two groups (*p* < 0.05). Hence, an analysis of covariance (ANCOVAs) was performed on each component using age as a covariable to compare participants with and without EDS. F- and p-values using the significant covariate are reported. Effect sizes (ES) were measured using the partial eta squared. Results were considered significant when p < 0.05.

In *Study 2*, for cortical surface analysis, general linear models (GLM) were built at each vertex to investigate the association between ESS and thickness, surface area, and volume of the cortical surface, controlling for the effects of age, sex, and PD duration. Since dosage of DA receptor agonists and PD duration were strongly correlated (*r* = 0.58, *p* = 0.001), only PD duration was included. Total intracranial volume was also added as a covariate for cortical surface area and volume analyses^75^. We also performed complementary analyses to investigate the presence of thickness, surface area, and volume differences between PD participants with and without EDS using the same covariates. Clusters were considered significant when *p* < 0.05 based on a Monte Carlo simulation approach.

For subcortical shape analysis, GLM were also used to study at each vertex the association between ESS and surface displacement in the 14 structures while controlling for age, sex and PD duration. Total intracranial volume was not added as a covariate since surface meshes were registered to the MNI152 space. Non-parametric permutation inference was performed using randomize with 10,000 permutations and a threshold-free cluster enhancement (TCFE) approach^76^. Clusters were considered significant when *p* < 0.05 after correction for family-wise error. The subcortical surface differences between groups were also investigated as complementary analyses using the same covariates.

Multiple regressions of ESS on VBM and DBM-derived brain measurements were used to predict whole-brain regional gray matter volume (GMV) using CAT12 version 12.7 for SPM12 under MATLAB R2018b (smoothing at 8 mm). All analyses were corrected for age, sex, and PD duration and total intracranial volume was added for VBM analyses. A TCFE was applied, and statistical significance was set at *p* < 0.05 corrected for multiple comparisons using false-discovery rate (FDR). Additionally, we performed t-tests for complementary between-group comparisons (PD with and without EDS) using the statistical module in CAT12.

### Supplementary information

Supplementary information

## Data Availability

The data supporting the findings of this study are available from the corresponding author upon reasonable request by any qualified investigator and in compliance with the specifications of the institutional ethics committees. Note that data sharing was not included in the informed consent signed by the patient, so approval from institutional ethics committees is required.

## Code availability

The underlying code for this study is not publicly available but may be made available to qualified researchers upon request to the corresponding author (see data availability).
