# Supplementary material for: Clinical symptoms and neuroanatomical substrates of daytime sleepiness in Parkinson’s disease
Source: NPJ Parkinsons Dis. 2024 Aug 9;10:149. doi: 10.1038/s41531-024-00734-x (PMC11316005; doi:10.1038/s41531-024-00734-x)
Supplement: Supplementary file 1 — Supplementary information [file 41531_2024_734_MOESM1_ESM.pdf]

Clinical Symptoms and Neuroanatomical Substrates of Daytime Sleepiness in Parkinson's Disease

Thaïna Rosinvil,[1-3] Ronald Postuma,[1,4] Shady Rahayel,[1,5] Amélie Bellavance,[2] Véronique Daneault[1-3], Jacques Montplaisir,[1,6] Jean-Marc Lina,[1,7, 8,] Julie Carrier[1-3]*, Jean-François Gagnon[1,9]*

[1]Center for Advanced Research in Sleep Medicine, CIUSSS-NÎM – Hôpital du Sacré-Coeur de Montréal, Montreal, Quebec, Canada
[2]Department of Psychology, Université de Montréal, Montreal, Quebec, Canada
[3]Research Center, Institut Universitaire de gériatrie de Montréal, Montreal, Quebec, Canada
[4]Department of Neurology, Montreal Neurological Institute, McGill University, Montreal, Quebec, Canada
[5]Department of Medicine, Université de Montréal, Montreal, Quebec, Canada
[6]Department of Psychiatry, Université de Montréal, Montreal, Quebec, Canada
[7]Department of Electrical Engineering, École de Technologie Supérieure, Montreal, Quebec, Canada
[8]Centre de Recherches Mathématiques, Université de Montréal, Montreal, Quebec, Canada
[9]Department of Psychology, Université du Québec à Montréal, Montreal, Quebec, Canada

[*]Corresponding authors:

Jean-François Gagnon, PhD
Centre for Advanced Research in Sleep Medicine, Hôpital du Sacré-Cœur de Montréal
5400 boulevard Gouin Ouest
Montreal, Quebec, Canada, H4J 1C5
Email: gagnon.jean-francois.2@uqam.ca

Julie Carrier, PhD
Centre for Advanced Research in Sleep Medicine, Hôpital du Sacré-Cœur de Montréal
5400 boulevard Gouin Ouest
Montreal, Quebec, Canada, H4J 1C5
Email: julie.carrier.1@umontreal.ca
Tel: 514-338-2222, ext. 3124, Fax: 514-338-2694

Supplementary Table 1. *Cognitive testing, variables and normative data.*

Cognitive domains and neuropsychological tests	Variables and normative data
**Attention**	
Digit Span[a]	Scaled score[a]
Trail Making Test, part A[b]	Time, seconds[c]
Stroop Color Word Test (modified version)[d]	Interference – Naming (time or number of errors)[r]<br>Interference – Word Reading (time)[r]
**Executive functions**	
<u>Trail Making Test, part B</u>[b]	Time, seconds[c]
Stroop Color Word Test (modified version)[d]	Flexibility – Interference (time or number of errors)[r]
Semantic (animals, fruits/vegetables) and phonetic (P, F, L in French; F, A, S in English) verbal fluencies[e]	Number of words (1 min)[f,g]
**Episodic memory**	
Rey Auditory Verbal Learning Test[h]	Sum of trials 1-5, list B, immediate recall, delayed recall, recognition[i]
**Visuo-spatial**	
Copy of the Rey Complex Figure Test[j,k]	Score/36 (40-68 years old,[l] >69 years old[m])
Block Design[a]	Scaled score[a]
<u>Bells test</u>[n]	Number of omission[n]
**Language**	
Boston Naming Test[o]	Score/30[s]
Vocabulary[a]	Scaled Score[a]
Mini Mental State Examination, language items[p]	Score/8[t]

Underlined tests were included in the Principal Component Analysis (PCA) of *Study 1*. Normative data used to establish performances can be found in the corresponding variable references.

[a]Wechsler D. Wechsler Adult Intelligence Scale, 3rd Ed. San Antonio TX: Harcourt Brace & Company; 1997.
[b]Army Individual Test Battery: Manual of Directions and Scoring. Washington DC: War Department, Adjutant General's Office; 1944.
[c]Tombaugh TN. Trail Making Test A and B: Normative data stratified by age and education. Arch Clin Neuropsychol 2004; 19: 203-14.
[d]Bohnen N, Jolles J, Twijnstra A. Modification of the Stroop color word test improves differentiation between patients with mild head injury and matched controls. Clin Neuropsychol 1992; 6: 178-84.
[e]Benton AL, Sivan AB, Hamsher K deS, Varney NR, Spreen O. Contributions to Neuropsychological Assessment: A Clinical Manual. 2nd Ed. New York: Oxford University Press; 1994.
[f]Lucas JA, Ivnik RJ, Smith GE, Bohac DL, Tangalos EG, Graff-Radford NR, et al. Mayo's older Americans normative studies: category fluency norms. J Clin Exp Neuropsychol 1998; 20: 194-200.
[g]Tombaugh TN, Kozak J, Rees L. Normative data stratified by age and education for two measures of verbal fluency: FAS and animal naming. Arch Clin Neuropsychol 1999; 14: 167-77.
[h]Rey A. L'examen clinique en psychologie. Paris: Presses Universitaires de France; 1964.
[i]Schmidt M. Rey Auditory-Verbal Learning Test. Los Angeles: Western Psychological Services; 1996.
[j]Rey A. L'examen psychologique dans les cas d'encéphalopathie traumatique. Arch Psychol 1941; 28: 286-340.
[k]Osterrieth PA. Le test de copie d'une figure complexe: contribution à l'étude de la perception et de la mémoire. Arch Psychol 1944; 30: 286-356.
[l]Speen O, Strauss E. A Compendium of Neuropsychological Tests: Administration, Norms, and Commentary. New York: Oxford University Press; 1991.
[m]Machulda MM, Ivnik RJ, Smith GE, Ferman TJ, Boeve BF, Knopman D, et al. Mayo's Older Americans Normative Studies: Visual Form Discrimination and Copy Trial of the Rey-Osterrieth Complex Figure. J Clin Exp Neuropsychol 2007; 29: 377-84.
[n]Gauthier L, Dehaut F, Joanette Y. The Bells Test: a quantitative and qualitative test for visual neglect. Int J Clin Neuropsychol 1989; 22: 49-54.
[o]Kaplan E, Goodglass H, Weintraub S. Boston Naming Test. 2nd Ed. Philadelphia: Lippincott Williams & Wilkins; 2001.
[p]Folstein MF, Folstein SE, McHugh PR. "Mini-mental state." A practical method for grading the cognitive state of patients for the clinician. J Psychiatr Res 1975; 12: 189-98.

Supplementary Table 2. *Variable list included in Multiple Imputation procedure and % of missing values.*

Apart from the executive control and processing speed measure for which we took both raw-score and z-score, raw-score items of selected variables were used in a data augmentation approach to Multiple Imputation (MI).[1] A total of 50 imputations were performed and 100 iterations per imputation were computed for convergence. This procedure amounts to alternate the simulation of missing data and the parameters estimation to converge in distribution for the missing data. Of note, we included in the MI procedure sufficient variables that were predictive of missing values (disease and motor severity and motor status, LEDD and DA agonist, expressed in LEDD, etc.) in addition to the ones we considered in further analyses (e.g., age, sex, RBD status).

**Included in MI and PCA**	**Items completed**	**Missing items**	**% of missing data**	**Included in MI only**	**Items completed**	**Missing items**	**% of missing data**
PD duration, y	77	0	0.0	PD duration, y	77	0	0.0
**DA agonist, LEDD mg**	**75**	**2**	**2.6**	**LEDD, mg**	**76**	**1**	**1.3**
UPDRS- III “on” score	77	0	0.0	**Hoehn & Yahr stage**	**76**	**1**	**1.3**
**BDI-II score**	**75**	**2**	**2.6**	ESS score	77	0	0.0
**BAI score**	**75**	**2**	**2.6**	Age, y	77	0	0.0
**ISI score**	**75**	**2**	**2.6**	Education, y	77	0	0.0
Sleep latency (min)	77	0	0.0	Sex, male n (%)	77	0	0.0
Sleep efficiency (%)	77	0	0.0	RBD, n (%)	77	0	0.0
N2-N3 sleep (%)	77	0	0.0	**TMT B, time (z score)**	**75**	**2**	**2.6**
**AHI**	**76**	**1**	**1.3**				
Mean O_2 saturation (%)	77	0	0.0				
MMSE, score	77	0	0.0				
**TMT B, time**	**72**	**5**	**6.5**				
Bells Test, number of omissions	77	0	0.0				
**Valid N (listwise)**	**66**	**11**	**14.3**				

Note. Variables in bold contain missing data. Valid N (listwise) = 66. Abbreviations: PD = Parkinson's disease. DA= Dopamine. LEDD = Levodopa Equivalent Daily Dosage. UPDRS-III = Unified Parkinson’s Disease Rating Scale, Part III. BDI-II = Beck Depression Inventory second edition. ESS = Epworth Sleepiness Scale. BAI = Beck Anxiety Inventory. ISI = Insomnia Severity Index. RBD = Rapid Eye Movement Sleep Behavioral Disorder. TMT B = Trail Making Test, part B. AHI = Apnea-Hypopnea Index.

1. Schafer JL. Analysis of Incomplete Multivariate Data. New York, NY: Chapman and Hall/CRC, 1997.
